# Variations of Network Centralities Between Playing Positions in Favorable and Unfavorable Close and Unbalanced Scores During the 2018 FIFA World Cup

**DOI:** 10.3389/fpsyg.2019.01802

**Published:** 2019-08-06

**Authors:** Filipe Manuel Clemente, Hugo Sarmento, Gibson Moreira Praça, Pantelis Theodoros Nikolaidis, Thomas Rosemann, Beat Knechtle

**Affiliations:** ^1^School of Sport and Leisure, Polytechnic Institute of Viana do Castelo, Melgaço, Portugal; ^2^Instituto de Telecomunicações, Delegação da Covilhã, Covilhã, Portugal; ^3^Research Unit for Sport and Physical Activity, Faculty of Sport Sciences and Physical Education, University of Coimbra, Coimbra, Portugal; ^4^Centro de Estudos em Cognição e Ação, Soccer Science Center, Departamento de Esportes, Escola de Educação Física, Fisioterapia e Terapia Ocupacional, Universidade Federal de Minas Gerais, Belo Horizonte, Brazil; ^5^Exercise Physiology Laboratory, Nikaia, Greece; ^6^Institute of Primary Care, University of Zurich, Zurich, Switzerland; ^7^Medbase St. Gallen Am Vadianplatz, St. Gallen, Switzerland

**Keywords:** graph theory, social network analysis, observational analysis, association football, performance analysis, notational analysis

## Abstract

The purpose of this study is twofold: (i) analyze the variations of network centralities between close (difference of goals equal to one) and unbalanced (difference of goals equal to or greater than two) scores; and (ii) compare the centrality levels between playing positions. The passing sequences that occurred during the 64 matches played by the 32 national teams that participated in the 2018 FIFA World Cup were analyzed and coded. The network centralities of degree prestige and degree centrality were calculated based on the weighted adjacency matrices built from the passing sequences. The results reveal that higher degree centralities of midfielders occurred in unfavorable (lost) unbalanced scores (*p* = 0.046; ES (effect size) = 0.472). Moreover, in favorable (won) matches the higher values of degree centrality of central defenders (*p* = 0.014; ES: 0.458) and defensive midfielders (*p* = 0.004; ES: 0.715) were also found in unbalanced scores. The comparisons between positions revealed that the highest and significant degree prestige levels were found in defensive midfielders in both close (12.10%) and unbalanced scores (10.95%). In conclusion, it is possible to observe that winning by an unbalanced score significantly increased the centrality levels of the wingers and forwards in comparison to close scores. Moreover, it was also found that independent of the final score or the unbalanced score level, the defensive midfielders were the most prominent or recruited players during the passing sequences.

## Introduction

The game of soccer allows the observation of two main types of relationships: (i) a network process between teammates aiming to synchronize the different individual behaviors and optimize the collective organization of the team; and (ii) a rapport of strength, that results from the interactions between two teams to beat each other in a dynamic system (Gréhaigne et al., 2011). Both relationships can be observed, analyzed and interpreted using different types of methodological approaches and using distinct techniques (Sarmento et al., 2017). Thus, the systematic observation may provide qualitative and/or quantitative information helping to build a deep understanding about the game and the intrinsic dynamics (Barreira et al., 2014; Sarmento et al., 2014).

Usually in match analyses conducted on soccer, there is a strong tendency to code, collect and report evidence about the variation of different performance indicators (e.g., successful passes, shots, recoveries) between different contextual factors (e.g., match status, location of the match, competitive level of the opponents, zone of the actions) (Lago-Peñas and Lago-Ballesteros, 2011; Tenga et al., 2017). However, some other performance indicators, such as tactical behavior (Barreira et al., 2013) or collective patterns based on position data (Clemente et al., 2017; Memmert et al., 2017), can be analyzed based on different contextual factors. Each derived piece of information presents its strong and weak points, i.e., the performance indicators based on notational analysis provide important information about the final outcomes of the match, although without considering the mechanisms and processes that justified such outcomes (Vilar et al., 2012). On the other hand, the observational analysis that allows the determination of the tactical behaviors and the position data analysis that provides information about the collective patterns of interactions also present limitations in establishing a connection between the behavior and the final outcome. For that reason, all the approaches present possibilities and threats.

In a mixed approach, the social network analysis (SNA) applied to team sports has proposed a new way to interpret the outcomes of the match (Duch et al., 2010; Grund, 2012). The process is clearly based on traditional notational analysis, however, allows computing and quantifying the interactions between teammates in order to extract some interpretation of how the nodes (players) are connected (Mclean et al., 2018). Despite having critics, namely because the SNA does not consider the spatio-temporal relationship or the tactical behavior associated, this method can provide interesting information about the overall relationship of the teammates in specific circumstances or contextual scenarios. The SNA applied to team sports follows the original principle of determining networks: nodes (players) connected by edges (a performance indicator) (Clemente et al., 2016b).

Using the pass as the linkage indicator in the network (i.e., edges) it has been possible to identify some evidence about the centrality levels of players during soccer matches (Duch et al., 2010; Peña and Touchette, 2012; Clemente et al., 2015b). In the case of an analysis carried out during the 2014 FIFA World Cup, it was possible to conclude that midfielders were the most prominent players in the interactions between teammates during passing sequences (Clemente et al., 2015b). Similar evidence was found in the study conducted on 2010 FIFA World Cup, also reporting that external and central defenders presented greater levels of prominence than forwards during the passing sequences (Peña and Touchette, 2012). Naturally, this evidence reported on both FIFA World Cups (2010 and 2014) were extracted from all passing sequences. However, in the specific case of passing sequences that resulted in goals or shots it was possible to observe that the forwards presented the greatest levels of network centralities (Clemente et al., 2016a). Moreover, in another analysis that characterizes only the passing sequences during counter-attack, the great prominence of forward players in comparison to defenders was observed (Malta and Travassos, 2014), thus suggesting that the network centralities are dependent on the context and the type of analysis conducted.

Considering the type of analysis, there is a lack of evidence about the influence of specific contextual factors in the studies that compared the network centrality levels of playing positions, namely considering the winning/losing factor and, most of all, the balance levels of the final scores. As one of the well-known contextual factors that may lead to different tactical behaviors and interactional processes between teammates, we hypothesize that network centralities may be different based on distinct contextual factors. Taking this rationale in mind, the unique study that tested the network levels between close scores (difference of one goal) and unbalanced scores (difference of two or more goals) revealed that the dyadic reciprocity levels of the players increased in winners during unbalanced matches and that total arcs and density were slightly greater in winners of close matches (Clemente, 2018). Those measures were related with general properties of the network and did not provide information about the influence of such contextual factors in the variations of centralities between playing positions. However, a better understanding about the mechanisms that may influence the prominence levels (centralities) of the players is required, mainly to identify if some patterns of interactions are sensitive to great fluctuations in the match score. Based on those reasons, the aim of this study were twofold: (i) analyze the variations of network centralities between close (difference of goals equal to one) and unbalanced (difference of goals equal to or greater than two) scores; and (ii) compare the centrality levels between playing positions. We hypothesize that network centralities will be different between the types of scores and that forward players will increase in centrality during the won matches in unbalanced conditions.

## Materials and Methods

### Sample

This study coded the passing sequences of the 64 matches of the 2018 FIFA World Cup. Therefore, all the 32 national teams were observed and included in the analysis process. The passing sequences were coded and included in the analysis after testing the intra- and inter-reliability level of the expert observers. The weighted adjacency matrices built based on the passing sequences were then treated for the subsequent network analysis.

### Ethics Statement

The study was approved by the local ethical committee (Polytechnic Institute of Viana do Castelo, School of Sport and Leisure) with the code IPVC-ESDL09052018. There is no contact or intervention with the players and the process was exclusively made based on observation.

### Study Design

This study followed a cross-sectional observational design. All the passing sequences made by each team during each entire match of the 2018 FIFA World Cup were observed, coded and transformed in weighted adjacency matrices. Those matrices were then treated and the degree prestige and degree centrality (network measures) were calculated considering the playing positions of the players: (i) goalkeeper (GK); (ii) external defenders (ED), players that act as defenders in the side of the field; (iii) central defenders (CD), players that acts as defenders in the central region of the field; (iv) defensive midfielders (DMF), players that acts as midfielders in a region closer to the central defenders; (v) midfielders (MF), players that acts in the middle of the field linking the defensive and attacking players; (vi) wingers (W), players that act in forward and side regions; and (vii) forwards (FW), players that acts in the middle of attacking regions. The variations of degree prestige and centrality between playing positions were tested considering the unbalance level of the final score. Based on such options, we have excluded the draw situations that did not provide the same score (losing or winning) that can be comparable between unbalanced and balanced games. The matches with a final score difference of one goal were considered close scores and those with two goals or more of difference as unbalanced scores. The classification of balanced vs. unbalanced score was exclusively made considering the final score (end of the match). This definition of balanced vs. unbalanced score was used in a previous study on soccer (Clemente, 2018).

### Observation, Data Codification and Production of Weighted Adjacency Matrices

Two expert observers (sport scientists with more than 5 years of experience on soccer) were recruited to observe and code the passing sequences of all matches of the 2018 FIFA World Cup. All the successful passes between two teammates were considered to include in the sample. Those observers were tested for their reliability levels following a pre-post pilot study design using a total of seven matches of the competition (10.94% of the all matches). The pre-post analysis was interspaced by a 20-day interval period aiming to test the reliability level of the observers. The process was made before the full-data being collected, aiming to ensure the desirable level of reliability. The results obtained from the pilot study revealed an average of intra-class correlation level of 0.97 (excellent reliability) for the case of intra-observer analysis and an average of 0.91 (excellent reliability) for the case of inter-observer analysis. The values obtained revealed that the reliability level of the observers was enough to follow through with the data collection (Koo and Li, 2016).

After confirmation of the reliability level of the observers to code the passes between teammates, all the matches were observed, coded and treated following a network analysis process. The players were first coded by the playing position in the pitch and even in the case of replacements or changes during the match, the aim was to analyze the centrality levels of positions and not the specific players (Clemente et al., 2015b; Mendes et al., 2018).

**FIGURE 1:**
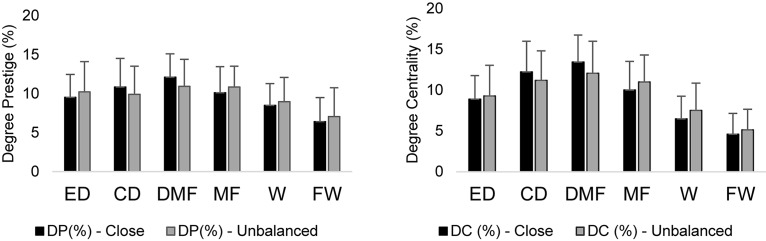
Example of codification and building of a weighted adjacency matrix.

Each passing sequence was converted in a weighted adjacency matrix (Figure 1). The passing sequence was considered by the uninterrupted sequence of passes between teammates with a minimum number of passes of two and with an undefined maximum (Clemente et al., 2015a). The passing sequence stopped in the case of a lost ball (caused by a recovery or interception of the opponents, loss of control, fault or a shot). The number of passing sequences per team varied from 52 to 103 during the matches. The direction and number of passes between playing positions were defined as the criteria to build the weighted adjacency matrix. Therefore, a pass from player A to player B was different from a pass from player B to player A (direction was considered). Moreover, the number of passes in the same direction was also considered. Thus, weighted digraphs were created based on this approach. The sum of all passing sequences during the match resulted in a final weighted adjacency matrix of that match. The specific methodology followed previous works in the field of network analysis on soccer match analysis (Clemente et al., 2015a, 2016c). Only the regular time of the match was analyzed, thus no extra-time (in the case of draws) was included. The weighted adjacency matrices were standardized based on the time of the players on the pitch.

### Network Analysis

The weighted adjacency matrices of all matches were imported into Social Network Visualizer software (version 2.4., Dimitris Kalamaras, Greece). This free software allows converting weighted adjacency matrices into networks and calculating the centrality levels of the nodes (players). For this study, the calculation of the standardized degree prestige and degree centrality was made.

#### Degree Prestige

This network measure quantifies the inbound links that a specific player received from his teammates (Clemente et al., 2016c) and, for that reason, a higher level of degree prestige means that the player is more often engaged by his teammates to participate in the passing sequences. The standardized value of degree prestige (DP’) was multiplied by 100 to have the final relative DP (DP%). The algorithm can be observed in previous works (Clemente et al., 2016c):

(1) $$D\,P_{D-i\,n}^{w}=k_{i}^{w}=\sum_{j=1}^{n}a_{j\,i},$$

in which *a*_*ji*_ can be considered the elements of the weighted adjacency matrix of a *G* with a *n*_*i*_ as vertex.

#### Degree Centrality

The network measure of degree centrality represents the overall level of connection of a player with the teammates, considering the number of outbounds, thus a higher level of degree centrality suggests that the player contributed more often to the passing sequence by executing more passes (Clemente et al., 2015b). The standardized degree centrality (DC’) was multiplied by 100 to have the final relative DC (DC%). The algorithm can be observed in previous works (Clemente et al., 2016c):

(2) $$D\,C_{D-o\,u\,t}^{w}\,\left(n_{i}\right)=k_{i}^{w-o\,u\,t}=\sum_{j=1}^{n}a_{i\,j},$$

in which *a*_*ji*_ can be considered the elements of the weighted adjacency matrix of a *G* with a *n*_*i*_ as vertex

### Statistical Procedures

The data was presented in form of text, tables and figures with means and the standard deviation (SD). The variations of centralities between playing positions (GK, ED, CD, DMF, MF, W and FW) and type of final score (close or unbalanced scores) were tested with a univariate MANOVA and one-way ANOVA with Tukey HSD *post hoc* test after confirmation of the normality and homogeneity assumptions of the sample. The partial eta squared tested the effect size (ES) of the univariate MANOVA. The statistical procedures were executed in the SPSS (version 24.0, IBM Statistics, United States) for a *p*-value < 0.05. Moreover, the Cohen *d* tested the ES of the pairwise comparisons between playing positions. The following scale was used to determine the magnitude of ES for the case of Cohen *d* (Batterham and Hopkins, 2006): 0.0−0.2, trivial effect; 0.2−0.6, small effect; 0.6−1.2, moderate effect; 1.2−2.0, large effect; and >2.0, very large effect.

## Results

### Close vs. Unbalanced Scores

The univariate MANOVA tested the interactions between factors revealing significant interactions in the pair difference of goals^∗^playing position (*p* = 0.001; ES: 0.028) and final score^∗^playing position (*p* = 0.008; ES: 0.20) for the case of degree prestige, however, no significant interaction were found in the pair difference of goals^∗^ final score (*p* = 0.775; ES: 0.000). In the case of degree centrality were found significant interactions in the pairs difference of goals^∗^playing position (*p* = 0.001; ES: 0.029) and final score^∗^playing position (*p* = 0.036; ES: 0.016), however, no significant interactions were found in the pair differences of goals^∗^final score (*p* = 0.561; ES: 0.000).

Comparisons of network centralities within playing positions and between close and unbalanced scores can be observed in Table 1. It was observed that in lost matches the degree centrality of midfielders was higher in unbalanced scores than in close (difference in means (dif): 1.68; *p* = 0.046; ES = 0.472, *small effect*). In won matches it was observed that the degree centralities were higher in close scores for the cases of central defenders (dif: 1.59; *p* = 0.014; ES: 0.458, *small effect*) and defensive midfielders (dif: 2.51; *p* = 0.004; ES: 0.715, *moderate effect*). Similar evidence was found for the case of degree prestige in central defenders (dif: 1.40; *p* = 0.028; ES: 0.409, small effect) and defensive midfielders (dif: 2.28; *p* = 0.006; ES: 0.691, moderate effect). On the other hand, a significant increase of degree centralities in unbalanced scores among wingers (dif: 2.01; *p* = 0.002; ES: 0.650, moderate effect) and forwards (dif: 1.30; *p* = 0.030; ES: 0.524, small effect) was also observed in won matches. The degree prestige also increased in midfielders in the case unbalanced scores (dif: 1.48; *p* = 0.014; ES: 0.526, small effect).

**TABLE 1:** Descriptive statistics of network centralities between playing positions in won and lost contexts split by close and unbalanced final scores.

		**Close M(SD)**	**Unbalanced M(SD)**	**Dif (C−U)**	***p*-value**	**ES**	**Magnitude**
**Lost**							
GK	DP (%)	3.10(1.77)	3.24(1.85)	–0.14	0.785	–0.078	*Trivial*
	DC (%)	5.33(2.56)	5.67(2.48)	–0.34	0.634	–0.135	*Trivial*
ED	DP (%)	9.93(2.93)	10.92(4.14)	–0.99	0.158	–0.287	*Small*
	DC (%)	9.25(3.01)	9.70(3.60)	–0.46	0.488	–0.138	*Trivial*
CD	DP (%)	10.58(3.99)	10.09(3.75)	0.49	0.508	0.126	*Trivial*
	DC (%)	11.81(4.18)	11.31(3.58)	0.49	0.512	0.127	*Trivial*
DMF	DP (%)	11.63(2.67)	11.70(3.61)	–0.08	0.923	–0.023	*Trivial*
	DC (%)	13.23(3.29)	13.23(3.96)	–0.01	0.993	0.001	*Trivial*
MF	DP (%)	9.90(3.49)	10.80(2.74)	–0.90	0.227	–0.283	*Small*
	DC (%)	9.63(3.64)	11.30(3.41)	–1.68	0.046^∗^	–0.472	*Small*
W	DP (%)	8.83(3.06)	8.32(2.85)	0.51	0.414	0.172	*Trivial*
	DC (%)	6.64(2.76)	6.77(3.01)	–0.13	0.824	–0.045	*Trivial*
FW	DP (%)	6.27(3.22)	6.18(2.84)	0.09	0.908	0.029	*Trivial*
	DC (%)	4.77(2.96)	4.46(2.01)	0.31	0.637	0.119	*Trivial*
**Won**							
GK	DP (%)	3.26(1.15)	3.31(1.68)	–0.05	0.894	–0.036	*Trivial*
	DC (%)	5.79(2.13)	6.07(2.93)	–0.28	0.682	–0.114	*Trivial*
ED	DP (%)	9.16(2.94)	9.54(3.50)	–0.38	0.552	–0.120	*Trivial*
	DC (%)	8.56(2.78)	8.87(3.93)	–0.32	0.631	–0.096	*Trivial*
CD	DP (%)	11.11(3.35)	9.71(3.53)	1.40	0.028	0.409	*Small*
	DC (%)	12.65(3.32)	11.06(3.71)	1.59	0.014	0.458	*Small*
DMF	DP (%)	12.65(3.29)	10.37(3.31)	2.28	0.006	0.691^∗^	*Moderate*
	DC (%)	13.71(3.34)	11.20(3.72)	2.51	0.004	0.715^∗^	*Moderate*
MF	DP (%)	10.29(3.28)	10.94(2.64)	–0.65	0.377	–0.211	*Small*
	DC (%)	10.34(3.42)	10.62(3.21)	–0.28	0.722	–0.084	*Trivial*
W	DP (%)	8.23(2.51)	9.71(3.28)	–1.48	0.014	–0.526	*Small*
	DC (%)	6.33(2.81)	8.34(3.53)	–2.01	0.002	−0.650^∗^	*Moderate*
FW	DP (%)	6.54(3.01)	7.88(4.27)	–1.34	0.120	–0.374	*Small*
	DC (%)	4.45(2.18)	5.74(2.82)	–1.30	0.030^∗^	–0.524	*Small*

*Close (C): close scores (difference of goals equal to 1); Unbalanced (U): unbalanced scores (difference of goals ≥ 2); Dif (B-U): difference of the means (B-U); ES: effect size using the Cohen (*d*); ^∗^Significant at *p*-value < 0.05 and moderate effect size.*

### Differences Between Positions

**FIGURE 2:**
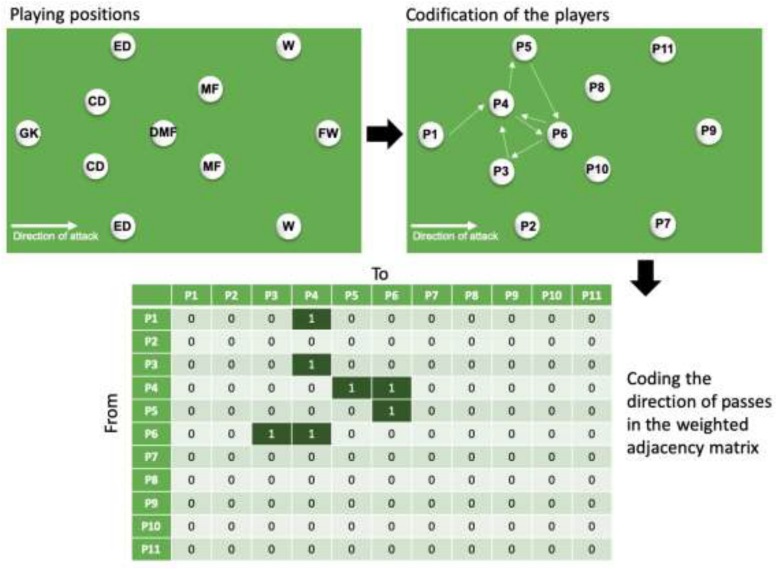
Descriptive statistics (mean and standard deviation) of degree prestige (DC) and degree centrality (DC) in close and unbalanced scores between playing positions. ED: external defender; CD: central defender; DMF: defensive midfielder; MF: midfielder; W: winger; FW: forward.

Descriptive statistics of network measures between playing positions in the case of close and unbalanced scores were tested (Figure 2).

Comparisons between positions revealed that the highest degree prestige levels were found in defensive midfielders in both close (12.10%) and unbalanced scores (10.95%). On the other hand, the forwards presented the lowest degree prestige in both close (6.41%) and unbalanced scores (7.06%). However, as can be observed in Table 2, the magnitude (ES) of differences between playing positions decreased from close to unbalanced matches (Table 2).

**TABLE 2:** Differences of degree prestige between playing positions in the cases of close and unbalanced scores.

	**Dif**.	***p*-value**	**ES**	**Magnitude**
**Close scores**				
ED vs. CD	–1.33	0.006	–0.397	*Small*
ED vs. DMF	–2.57	0.001	−0.865^∗^	*Moderate*
ED vs. MF	–0.59	0.798	–0.185	*Trivial*
ED vs. W	1.02	0.127	0.355	*Small*
ED vs. FW	3.11	0.001	1.039^∗^	*Moderate*
CD vs. DMF	–1.24	0.049	–0.360	*Small*
CD vs. MF	0.75	0.522	0.211	*Small*
CD vs. W	2.35	0.001	0.710^∗^	*Moderate*
**CD vs. FW**	**4.45**	**0.001**	**1.280^∗∗^**	***Large***
DMF vs. MF	1.99	0.001	0.623^∗^	*Moderate*
**DMF vs. W**	**3.59**	**0.001**	**1.247^∗∗^**	***Large***
**DMF vs. FW**	**5.69**	**0.001**	**1.869^∗∗^**	***Large***
MF vs. W	1.61	0.003	0.523	*Small*
MF vs. FW	3.70	0.001	1.142^∗^	*Moderate*
W vs. FW	2.10	0.001	0.720^∗^	*Moderate*
**Unbalanced scores**				
ED vs. CD	0.34	0.994	0.091	*Trivial*
ED vs. DMF	–0.71	0.897	–0.191	*Trivial*
ED vs. MF	–0.62	0.932	–0.182	*Trivial*
ED vs. W	1.26	0.222	0.358	*Small*
ED vs. FW	3.18	0.001	0.835^∗^	*Moderate*
CD vs. DMF	–1.05	0.532	–0.294	*Small*
CD vs. MF	–0.96	0.588	–0.292	*Small*
CD vs. W	0.92	0.557	0.270	*Small*
CD vs. FW	2.84	0.001	0.775^∗^	*Moderate*
DMF vs. MF	0.09	1.000	0.029	*Trivial*
DMF vs. W	1.97	0.018	0.603^∗^	*Moderate*
DMF vs. FW	3.89	0.001	1.080^∗^	*Moderate*
MF vs. W	1.89	0.020	0.642^∗^	*Moderate*
MF vs. FW	3.80	0.001	1.177^∗^	*Moderate*
W vs. FW	1.92	0.018	0.566	*Small*

*Close (C): close scores (difference of goals equal to 1); Unbalanced (U): unbalanced scores (difference of goals ≥ 2); Dif: difference of the means of playing positions (A-B); ES: effect size using the Cohen (*d*); ^∗^Significant at *p*-value < 0.05 and moderate effect size; ^∗∗^Significant at *p*-value < 0.05 and large effect size; ^∗∗∗^Significant at *p*-value < 0.05 and very large effect size; Highlighted in bold: large to very large differences.*

The greatest degree of centrality levels were verified in defensive midfielders in both close (13.45%) and unbalanced matches (12.08%). On the other hand, the lowest degree centralities were observed in forwards in both close (4.60%) and unbalanced matches (5.12%). However, similarly to the case of degree prestige, the magnitude of changes between playing positions decreased from close to unbalanced matches (Table 3).

**TABLE 3:** Differences of degree centrality between playing positions in the cases of close and unbalanced scores.

	**Diff**.	***p*-value**	**ES**	**Magnitude**
**Close scores**				
ED vs. CD	–3.36	0.001	−0.993^∗^	*Moderate*
**ED vs. DMF**	−**0.46**	**0.001**	−**1.489^∗∗^**	***Large***
ED vs. MF	–1.13	0.109	–0.356	*Small*
ED vs. W	2.41	0.001	0.851^∗^	*Moderate*
**ED vs. FW**	**4.29**	**0.001**	**1.546^∗∗^**	***Large***
CD vs. DMF	–1.20	0.077	–0.333	*Small*
CD vs. MF	2.23	0.001	0.608^∗^	*Moderate*
**CD vs. W**	**5.78**	**0.001**	**1.718^∗∗^**	***Large***
**CD vs. FW**	**7.66**	**0.001**	**2.258^∗∗∗^**	***Very large***
DMF vs. MF	3.43	0.001	1.004^∗^	*Moderate*
**DMF vs. W**	**6.98**	**0.001**	**2.320^∗∗∗^**	***Very large***
**DMF vs. FW**	**8.86**	**0.001**	**2.988^∗∗∗^**	***Very large***
MF vs. W	3.55	0.001	1.133^∗^	*Moderate*
**MF vs. FW**	**5.43**	**0.001**	**1.739^∗∗^**	***Large***
W vs. FW	1.88	0.001	0.695^∗^	*Moderate*
**Unbalanced scores**				
ED vs. CD	–1.89	0.006	–0.514	*Small*
ED vs. DMF	–2.79	0.001	−0.728^∗^	*Moderate*
ED vs. MF	–1.70	0.560	–0.478	*Small*
ED vs. W	1.78	0.021	0.496	*Small*
**ED vs. FW**	**4.17**	**0.000**	**1.262^∗∗^**	***Large***
CD vs. DMF	–0.90	0.724	–0.238	*Small*
CD vs. MF	0.19	1.000	0.054	*Trivial*
CD vs. W	3.67	0.001	1.045^∗^	*Moderate*
**CD vs. FW**	**6.06**	**0.001**	**1.864^∗∗^**	***Large***
DMF vs. MF	1.09	0.622	0.299	*Small*
**DMF vs. W**	**4.57**	**0.001**	**1.271^∗∗^**	***Large***
**DMF vs. FW**	**6.96**	**0.001**	**2.130^∗∗∗^**	***Very large***
MF vs. W	3.48	0.001	1.046^∗^	*Moderate*
**MF vs. FW**	**5.87**	**0.001**	**1.992^∗∗^**	***Large***
W vs. FW	2.39	0.001	0.796^∗^	*Moderate*

*Close (C): close scores (difference of goals equal to 1); Unbalanced (U): unbalanced scores (difference of goals ≥ 2); Dif: difference of the means of playing positions (A-B); ES: effect size using the Cohen (*d*); ^∗^significant at *p*-value < 0.05 and moderate effect size; ^∗∗^Significant at *p*-value < 0.05 and large effect size; ^∗∗∗^significant at *p*-value < 0.05 and very large effect size; Highlighted in bold: large to very large differences.*

## Discussion

The network centralities allows to better identify the prominence of each player in the passing sequences of a team, namely considering the direction and weight of the passes (Cintia et al., 2015). In the present study it was observed that the prominence levels of specific playing positions are relatively stable (with very few exceptions) independent of the type of score and the unbalanced level of the scores.

The first purpose of the study was to analyze the variations of centrality levels between favorable and unfavorable close and unbalanced scores within playing positions. The results revealed that in unfavorable results (lost) only the external defenders and defensive midfielders presented meaningful small increases of prominence levels in unbalanced matches, however, the remaining playing positions did not meaningfully change the prominence levels. In the other hand, in favorable (won) results there was two main type of evidence: (a) central defenders and defensive midfielders had meaningful greater values of degree prestige and degree centrality in close scores; (b) midfielders, wingers and forwards had meaningful greater values of degree centrality and degree prestige in unbalanced scores. Therefore, two main conclusions could be extracted from the results: (i) generally, independent from the differences of goals in unfavorable results, there are no meaningful differences in the degrees of centrality and prestige of the great majority of the playing positions; and (ii) won by one goal requires a meaningful greater participation of central defenders and defensive midfielders, however, won by an unbalanced score requires a meaningful increase in the centralities of the positions that occupy forward lines, namely midfielders, wingers and forwards.

The fact of central defenders and defensive midfielders increasing their participation in favorable close scores may result from the team strategy to keep the ball in zones of low pressure and more security than in forward regions may be what increases the possibility of non-success (Kite and Nevill, 2017). The style of play associated with indirect attack more often requires backward passes, thus likely increasing the time taken to reach the opposing goal and recruiting a greater participation of the players in the first and second thirds of the attacking building (Fernandez-Navarro et al., 2016). In fact, more successful teams seems to recruit more defenders through passing behavior, thus justifying the great levels of centralities of the defenders and defensive midfielders (Rein et al., 2017). On the other hand, in the contexts of favorable and unbalanced scores, the possible greater volume of direct attacks may justify the meaningful increases of degree prestige and centrality of midfielders, wingers and forwards. In fact, attacking process in forward zones seems to recruit the wings more often (Barreira et al., 2015) and this may explain the moderate increases of degree centrality of wingers in favorable unbalanced scores. Moreover, a study that tested the general network properties of winners and losers in close and unbalanced scores also revealed that in situations of favorable and unbalanced scores there was a likely moderate increase of dyad reciprocity (Clemente, 2018), thus suggesting an increase in the overall participation of the teammates during the passing sequences.

The present study also tested the variations of network centralities between playing positions. In terms of close scores, it was found that the degree prestige (inbound) was largely greater for central defenders and defensive midfielders than for wingers and forwards. Interestingly, the magnitude of the differences decreased in the case of unbalanced scores, despite revealing the same tendencies. The degree prestige can be considered an indicator of the overall prominence level of a player to be recruited by his teammates (Clemente and Martins, 2017). The results observed are in line with the majority of the studies that analyzed the prestige level of different playing positions during passing sequences (Duch et al., 2010; Peña and Touchette, 2012; Clemente et al., 2015b). In fact, it is reasonable to expect that the majority of the passes occur between the defensive players during general passing sequences starting from defensive regions, thus indirectly justifying the greater indegree centralities (Mendes et al., 2018).

In the case of degree centrality (outbound), larger differences were observed between playing positions than in the case of degree prestige, namely considering the comparisons of more defensive positions (external and central defenders and defensive midfielders) with forward players (wingers and the forwards). Forwards and wingers were clearly and meaningfully- less prominent than the remaining playing positions in both close and unbalanced scores. This suggests that the overall participation of these two positions in constructing the passing sequences and establishing relationships with their teammates is significantly smaller; however, this depends on the type of analysis. Naturally, in the case of counter-attacks or passing sequences that result in shots or goals, the rate of prominence may increase in forward players, based on previous research (Malta and Travassos, 2014; Clemente et al., 2016a). However, considering all the passing sequences, the contribution is naturally lower because all the types of attacks are included (indirect and direct attacks).

This study had some limitations. The passing sequences were not split by type of attack or final outcome and for that reason the results about the prominence level should be carefully interpreted. Moreover, the analysis of different team’s formations was not considered. Also, there is no information about the tactical behavior that explains the prominence levels observed. Based on those limitations, it is important for future studies to split the passing sequences by type (e.g., indirect or direct attack) and final outcome (e.g., lose the ball, shot, goal) and also add information about the pitch regions in which the passes occurred. The analysis of the formations and tactical behavior of the players should also be considered to provide a qualitative interpretation and to increase the holistic view about the dynamics that contribute to the final outcomes. Future studies should also consider other technical actions that may provide information about the interactions between team players. Moreover, an analysis that considers the spatio-temporal dimension should be considered to improve the understanding about the dynamics of the match. To do that, it may be important to add an analysis per period of time (e.g., 10 in 10 min) or per changes in specific moments (e.g., after scoring or suffering a goal).

Despite the study limitations, this study is, in the best of our knowledge, the first that compared the prominence level of different playing positions in favorable and unfavorable close and unbalanced scores. The results of this study allow coaches to identify that favorable unbalanced scores increases the overall centrality levels of wingers and forwards and this may represent a transfer for training scenarios or even to options to make during the matches. Moreover, the evidence that defensive players are more prominent in building the attack in the generality of the passing sequences should encourage adopting strategies to reduce the success of the opponent’s teams in their zone of comfort.

## Conclusion

Considering the comparisons of network centralities between close and unbalanced scores, the main evidence revealed that defensive midfielders and central defenders presented meaningful greater levels of centrality (inbound and outbound) in won close scores than in unbalanced. On the other hand, won unbalanced matches meaningfully increased the centrality levels of wingers and forwards. Regarding the second purpose of this study – to compare the variations of network centralities between playing positions – it was possible to observe that defensive midfielders were the most recruited and also the most contributing to the passing sequences and that the forwards and wingers presented the lowest values of participation in both close and unbalanced matches. As a conclusion, this study suggests that independent of the magnitude of difference in the final score, there are playing positions (the midfielders) that are relatively stable in the participation during passing sequences and other positions (the forward players) that increase in participation in favorable unbalanced scores.

## Data Availability

The datasets for this manuscript are not publicly available because data are available upon request from the first author FC: filipe.clemente5@gmail.com. Requests to access the datasets should be directed to FC: filipe.clemente5@gmail.com.

## Ethics Statement

The study was approved by the local ethical committee (Polytechnic Institute of Viana do Castelo, School of Sport and Leisure) with the code IPVC-ESDL09052018. There is not contact or intervention with the players and the process was exclusively made based on the observation.

## Author Contributions

FC conceived the study. FC, HS, and GP designed the study. FC collected, analyzed, and interpreted the data. FC, HS, GP, PN, TR, and BK drafted, revised and approved the final version.

## Conflict of Interest Statement

The authors declare that the research was conducted in the absence of any commercial or financial relationships that could be construed as a potential conflict of interest.
